# The Potential Use of Antibiotics Against Helicobacter pylori Infection: Biopharmaceutical Implications

**DOI:** 10.3389/fphar.2022.917184

**Published:** 2022-06-27

**Authors:** Amir Hossein Miri, Mojtaba Kamankesh, Antoni Llopis-Lorente, Chenguang Liu, Matthias G. Wacker, Ismaeil Haririan, Hamid Asadzadeh Aghdaei, Michael R. Hamblin, Abbas Yadegar, Mazda Rad-Malekshahi, Mohammad Reza Zali

**Affiliations:** ^1^ Department of Pharmaceutical Biomaterials and Medical Biomaterials Research Center, Faculty of Pharmacy, Tehran University of Medical Sciences, Tehran, Iran; ^2^ Foodborne and Waterborne Diseases Research Center, Research Institute for Gastroenterology and Liver Diseases, Shahid Beheshti University of Medical Sciences, Tehran, Iran; ^3^ Polymer Chemistry Department, School of Science, University of Tehran, Tehran, Iran; ^4^ Institute for Complex Molecular Systems, Eindhoven University of Technology, Eindhoven, Netherlands; ^5^ College of Marine Life Science, Ocean University of China, Qingdao, China; ^6^ Department of Pharmacy, National University of Singapore, Singapore, Singapore; ^7^ Basic and Molecular Epidemiology of Gastrointestinal Disorders Research Center, Research Institute for Gastroenterology and Liver Diseases, Shahid Beheshti University of Medical Sciences, Tehran, Iran; ^8^ Laser Research Centre, Faculty of Health Science, University of Johannesburg, Doornfontein, South Africa; ^9^ Gastroenterology and Liver Diseases Research Center, Research Institute for Gastroenterology and Liver Diseases, Shahid Beheshti University of Medical Sciences, Tehran, Iran

**Keywords:** *H. pylori*, antibiotic therapy, biopharmaceutical principles, pharmaceutical microbiology, pharmacokinetics, pharmacodynamics

## Abstract

*Helicobacter pylori* (*H. pylori*) is a notorious, recalcitrant and silent germ, which can cause a variety of debilitating stomach diseases, including gastric and duodenal ulcers and gastric cancer. This microbe predominantly colonizes the mucosal layer of the human stomach and survives in the inhospitable gastric microenvironment, by adapting to this hostile milieu. In this review, we first discuss *H. pylori* colonization and invasion. Thereafter, we provide a survey of current curative options based on polypharmacy, looking at pharmacokinetics, pharmacodynamics and pharmaceutical microbiology concepts, in the battle against *H. pylori* infection.

## Graphical Abstract

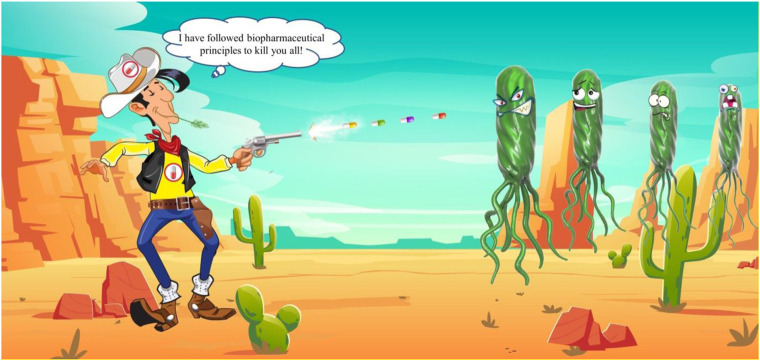

## Introduction

**FIGURE 1:**
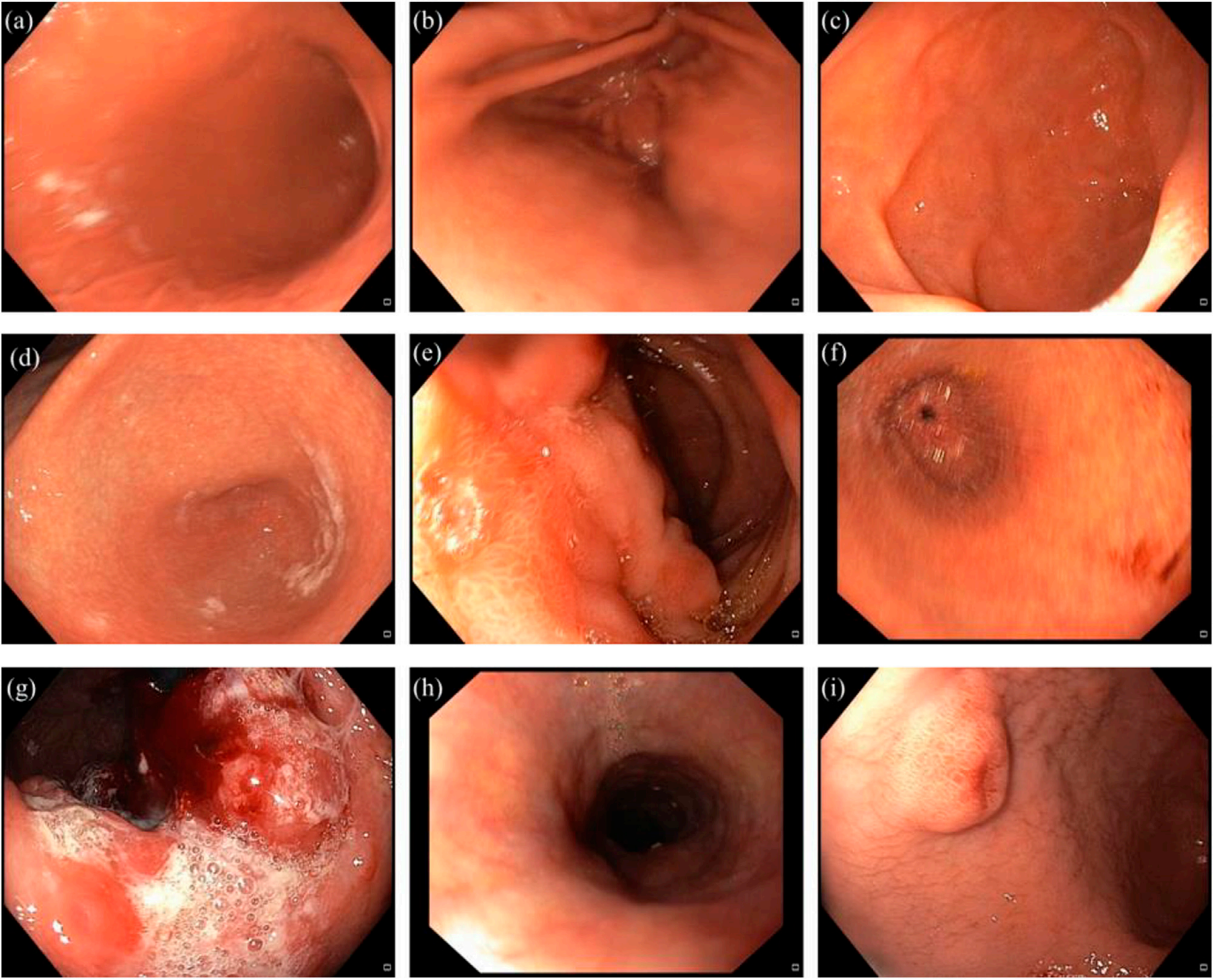
Representation of healthy stomach compartments, and different gastric disorders induced by *H. pylori*. **(a)** normal body. **(b)** normal antrum. **(c)** normal duodenum. **(d)** antral gastritis. **(e)** duodenal ulcer. **(f)** gastric ulcer. **(g)** gastric cancer. **(h)** esophagitis. **(i)** polypoid lesion in the antrum.

The Nobel Prize in medicine or physiology 2005 was jointly awarded to the Australian-born scientists, Robin Warren and Barry J. Marshall, for the discovery of *Helicobacter pylori* (*H. pylori*), as a fastidious Gram-negative microaerophilic spiral bacterium (Warren and Marshall, 1983; Arora et al., 2012). This germ is 2.5–5 μm long and 0.5–1.0 μm wide, with six unipolar sheathed flagella visible under electron microscopy (Goodwin and Armstrong, 1990; Geis et al., 1993; Zhao et al., 2014). In 1994, the international agency for research on cancer (IARC) classified *H. pylori* as a type І carcinogen and also declared that there was a strong relation between gastric cancer and this potentially deadly pathogen (Li et al., 2019). Over the years, *H. pylori* has been discovered to cause a variety of gastric diseases such as peptic ulcer, gastric ulcer and gastric cancer (Figure 1) (Hunt, 1996; Umamaheshwari and Jain, 2003; Hussein et al., 2008; Garza-González et al., 2014; Liu et al., 2021).

Moreover, *H. pylori* can be associated with some non-gastric diseases, such as cardiovascular, respiratory, neurologic, hematologic, dermatologic and allergic disorders (Gravina et al., 2018; Attaran et al., 2021). As one example, it was discovered that there was a correlation between *H. pylori* infection and problems in the digestive tract, such as abdominal pain and diarrhea in Coronavirus disease of 2019 (COVID-19) patients (Balamtekin et al., 2021). As time goes on, despite valuable experience in predicting the best treatment protocols for elimination of this pathogen from the human stomach, until now the treatment of *H. pylori* infection remains a major challenge for clinicians (Zhao et al., 2014). It has been reported that this bacterial pathogen has contributed to almost 75% of the gastric cancer burden worldwide (Amieva and Peek, 2016). Incredibly, in the United States the rate of infection remained consistent during the years 2000–2010, while the rate of eradication dramatically decreased (Thung et al., 2016; Gong and Yuan, 2018). Also the rate of incidence in developing and developed countries was 50.8% and 34.7%, respectively (Peng et al., 2018; Zamani et al., 2018). This incidence is maximum during childhood (11–16 years old), under poor socioeconomic conditions, and also with poor overall hygiene (Mishra et al., 2008; Arora et al., 2012; Mahmoudi et al., 2017). Regarding this bacterium, one of the key principles to take into account is its transmission routes. Despite some discrepancies in the exact mode of transmission (Ng et al., 2017), person-to-person is the major route of transmission (Jabbari Moghaddam et al., 2009; Eusebi et al., 2014). However, environmental transmission, such as consumption of polluted water, vegetables, animal products such as milk and meat contaminated with feces can transmit *H. pylori* into the human body (Vale and Vítor, 2010; Ghanbari et al., 2020). Unfortunately, this pathogen can survive in contaminated water and food products for long periods of time (Quaglia and Dambrosio, 2018; Ghanbari et al., 2020). In order to understand the emerging foodborne and waterborne diseases associated with *H. pylori*, Monno et al. (2019) investigated the relation between dietary habits and *H. pylori* infection status in Apulia (Southern of Italy). The authors reported that there was a significant relationship between *H. pylori* positivity and consumption of some foods, such as raw mussels and other molluscs, which had been extensively consumed by local people of that area. Despite older studies, which could not culture *H. pylori* from polluted water or food products, several pioneering microbiological studies confirmed that *H. pylori* could indeed be isolated and cultured from contaminated water and food (Hemmatinezhad et al., 2016; Ranjbar et al., 2016b; Ranjbar et al., 2016a). The current literature shows that there is a difference in the % prevalence of *H. pylori* infection in the world population, due to variations in socioeconomic and hygiene conditions (Hu et al., 2017; Saxena et al., 2020). Moreover, according to the World Health Organization (WHO), stomach cancer associated with *H. pylori* caused up to 0.5 million deaths in the world population (Arora et al., 2012). There are several comprehensive reviews which have discussed the pathogenesis and pharmacotherapy of *H. pylori* infection (Georgopoulos and Papastergiou, 2020; Alexander et al., 2021; Tshibangu-Kabamba and Yamaoka, 2021; Ansari and Yamaoka, 2022; Sousa et al., 2022). To our knowledge, there has been little attention paid to the potential use of antibiotics and other antimicrobial agents in terms of the biopharmaceutical implications. In this review, we first discuss *H*. *pylori* colonization and invasion in an easy-to-understand manner. Thereafter, we provide a survey of current curative options based on polypharmacy, looking at pharmacokinetics, pharmacodynamics and pharmaceutical microbiology concepts, in the battle against *H*. *pylori* infection.

## *H. pylori* Colonization and Invasiveness

**FIGURE 2:**
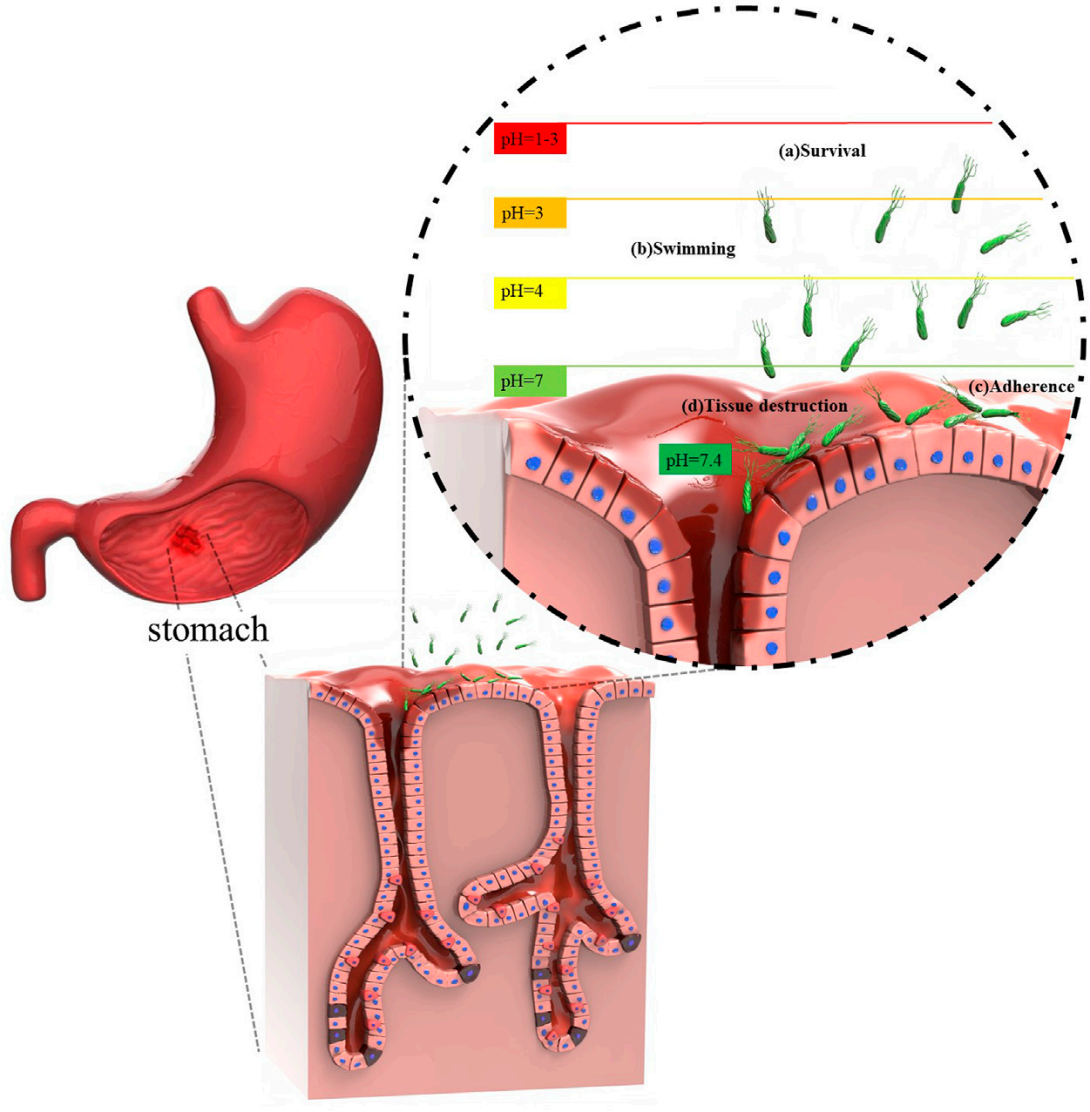
Sketch illustrating *H. pylori* invasion and colonization.

Generally *H*. *pylori* resides deeply in the antrum area of the human stomach where the viscous mucus layer protects the bacteria from the effects of the lower pH (Kusters et al., 2006; Zhao et al., 2014; Worku et al., 2015). Generally speaking, the steps of the *H. pylori*-invasion pathway can be categorized as four consecutive processes: 1) survival; 2) swimming; 3) adherence; 4) tissue destruction (Walton, 2016). The steps of *H. pylori* invasion are depicted in Figure 2.

**FIGURE 3:**
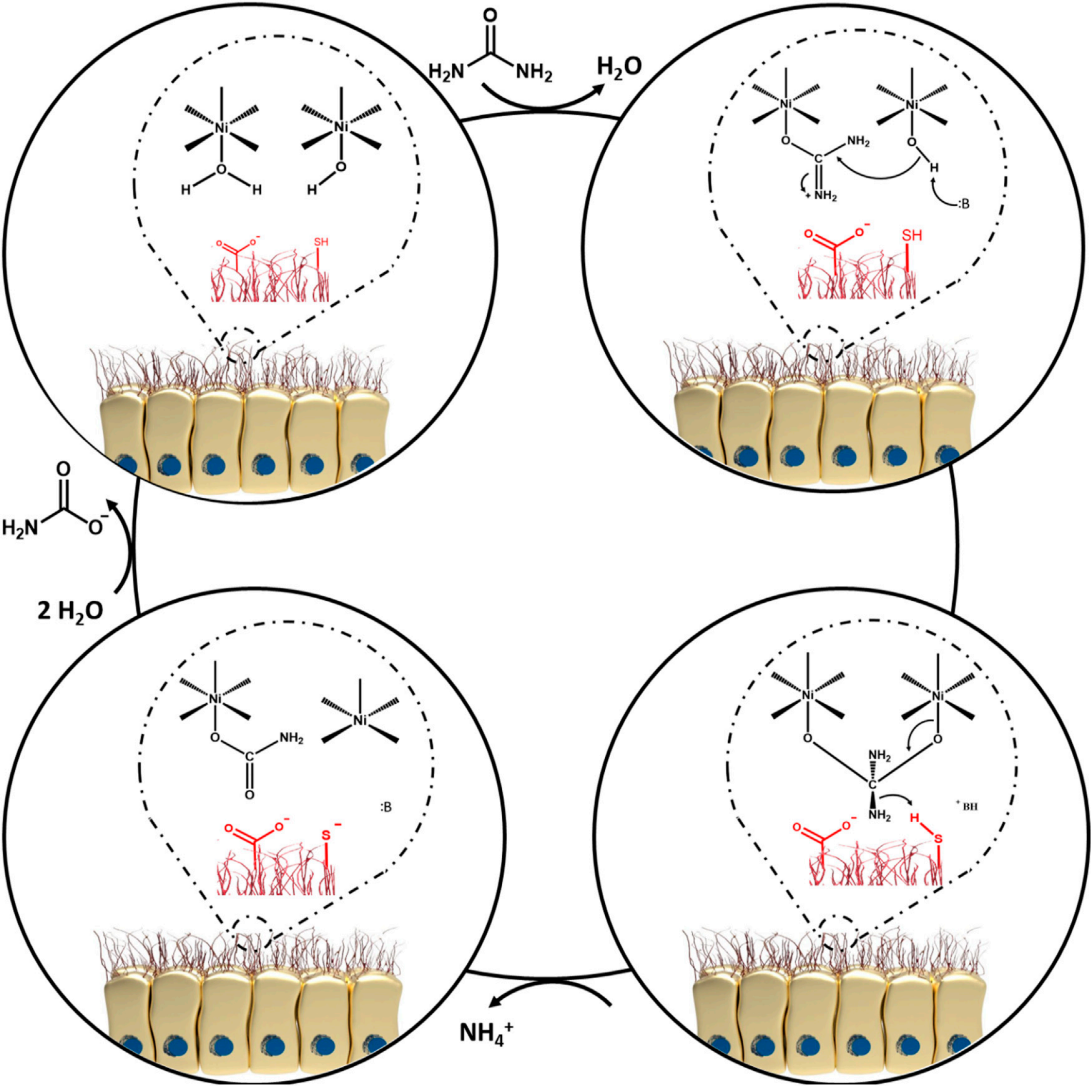
The proposed bioinorganic mechanism of urease enzyme activity.

Upon bacterial entry into the stomach, the obstacles standing in the way of *H. pylori* include, gastric acidity, motility, peristaltic movement of the stomach, and mucosal materials secreted by the glands of the epithelial cells. All these factors intrinsically operate against the pathogen to retard its colonization (Arora et al., 2012). However, it is also known that *H. pylori* is an adaptable germ and can escape from host defense mechanisms (Karkhah et al., 2019; Mao et al., 2021). Colonization of the gastric mucosa is made possible by exploiting a unique arsenal of weapons, including production of a potent urease enzyme, possessing rapid motility, and expressing a number of adherence molecules on its cell membrane (Arora et al., 2012). It should be pointed out that non-motile flagellated or non-flagellated *H. pylori* bacteria are less able (or even unable) to initiate cellular infection (Skene et al., 2007). In general, *H. pylori* colonization occurs in the gastric mucus layer close to the epithelial cells of the stomach, but the bacteria usually do not directly colonize the epithelial cells themselves (Arora et al., 2012; Zhao et al., 2014). However, it has been proposed that *H. pylori* bacteria can be localized within the intracellular or intercellular space of gastric epithelial cells (Necchi et al., 2007; Chu et al., 2010; Arora et al., 2012; Kao et al., 2016; Sit et al., 2020). The epithelial cells are shielded by a thick layer of mucus as a barrier against foreign invasion, therefore *H. pylori* has to employ its flagellae to penetrate towards the underlying epithelial cells (Walton, 2016; Charitos et al., 2021). In addition, a variety of outer membrane proteins are expressed by *H. pylori* strains, such as *H. pylori*-outer membrane protein (Hop) and Hop-related proteins (Hor) (Feige et al., 2018). The Hop protein category act as virulence factors, including blood-group antigen binding adhesion (BabA), sialic acid-binding adhesion (SabA), *H. pylori*-outer membrane protein Q (HopQ), adherence-associated lipoproteins A and B (AlpA and AlpB) which are responsible for adhesion to the gastric mucosal layer (Oleastro and Ménard, 2013; Salama et al., 2013; Feige et al., 2018). Indeed, these factors permit the bacteria to gain a foothold in mucosal niches (Couturier, 2013). During adhesion to the gastric mucosa, the urease enzyme metabolizes urea to ammonia and bicarbonate, elevating the pH towards neutral (Arora et al., 2012; Piscione et al., 2021; Serrano et al., 2021). Hence, *H. pylori* provides the appropriate conditions in the gastric mucus to survive, and develop gastric disease and malignancies (Arora et al., 2012; Piscione et al., 2021). Given this situation, some advanced investigations have focused on the bioinorganic mechanism of urease as a nickel-containing metalloenzyme (Dixon et al., 1980; Jabri et al., 1995; Karplus et al., 1997; Pearson et al., 1997). As a matter of fact, Ni is considered to be an essential co-factor for urease activity (Maier and Benoit, 2019; Mazzei et al., 2020; Maroney and Ciurli, 2021). One bioinorganic mechanism was proposed by Dixon et al. (1980). At the first step, water and hydroxyl-containing molecules are coordinated by one of the two active sites of Ni (І) or Ni (ІІ) ions (coordination involves binding of ligands to a central metal atom). At the beginning of this process, the water molecule is replaced with urea. At the second step, a nucleophilic attack occurs on the carbonyl C atom by Ni (ІІ) to produce a tetrahedral intermediate. At the third step, this intermediate becomes unstable at basic pH and is easily disintegrated. Next, a carbamate ester and ammonium cation are generated. Following this reaction, water molecules can easily cause the release of the carbamate ester, and finally all of the mentioned cycles are repeated again. Figure 3 illustrates the proposed mechanism.

**FIGURE 4:**
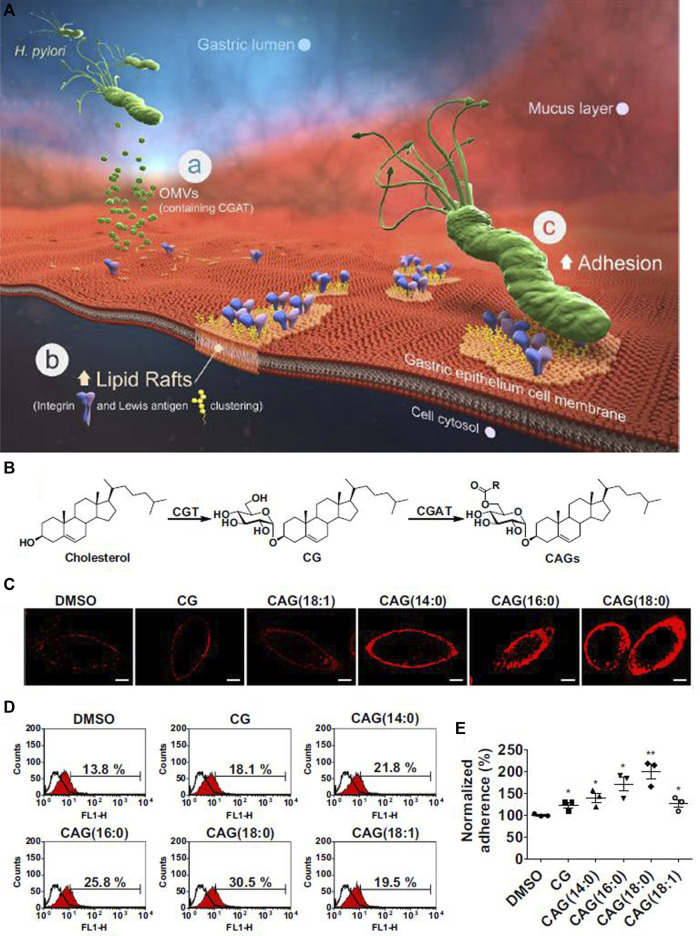
*H. pylori* attachment in the stomach. **(A)** The major steps of *H. pylori* attachment. **(a)** The secretion of OMV (outer membrane vesicle) to deliver CGAT (cholesteryl-D-glucopyranoside acyltransferase) and CGT (cholesterol α-glucosyltransferase) enzymes to human gastric cells. **(b)** The formation of lipid-raft clustering to facilitate bacterial adhesion. **(c)** Localization of integrin and Lewis antigens at the site of adhesion, leading to bacterial adhesion. **(B)** The scheme for production of CAG (6′-O-acyl-α-D-glucopyranoside). **(C)** Confocal images of CAG with different acyl side chains. **(D)** and **(E)** Effect of different acyl-side chains of CAG on the amount of *H. pylori* adherence to gastric epithelial cells. Reproduced from (Jan et al., 2020). Copyright 2020 with permission from “Springer Nature”.

In order to facilitate the adhesion process, some components of the bacterial type IV secretion system (T4SS) apparatus, such as CagI, CagL, CagY and CagA (Cag: Cytotoxin-associated gene) are able to interact with integrin β_1_ (Kwok et al., 2007; Backert et al., 2011; Salama et al., 2013). For more information on the T4SS system, interested readers are referred to the following review (Costa et al., 2021). The role of adhesion factors is apparent in the elegant work by Jan et al. (2020). In brief, the major concept involves the activity of the pathogenicity island 6′-O-acyl-α-D-glucopyranoside (CAG) in the bacteria, through hijacking of cholesterol from human gastric epithelial cells thereby forming a lipid-raft cluster, and promoting bacterial attachment to human gastric cells (Figure 4A). During the acquisition of cholesterol, the Glucosyltransferase (CGT) enzyme catalyzes the conversion of cholesterol to Cholesteryl α-D-glucopyranoside (CG). Afterward, further functionalization on the O6′ group of glucose of CG occurs by the action of the Cholesteryl α-D-glucopyranoside 6′-acyltransferase (CGAT) enzyme. CGAT can produce human lipid-containing CAGs with different fatty acid acyl side chains, including myristic acid (14:0), palmitic acid (16:0), stearic acid (18:0) or oleic acid (18:1), as shown in Figure 4B. Confocal microscopy has been used to visualize CAGs with different acyl side chains Figure 4C. Upon treatment with CG or CAG for 1 h, lipid rafts in AGS cells were labeled with AlexaFluor 594-conjugated cholera toxin subunit b (red fluorescence). The imaging revealed that the amount of bacterial adhesion was directly related to the presence of different acyl side chains on CAG (Figures 4D,E). This study showed that lipid-raft clustering with an increase in the length of the acyl-side chains of CAG, increased the delivery of CagA into gastric host cells.

The flagellar sheath of *H. pylori* acts as a shelter to protect against acidic stress conditions (Geis et al., 1993; Sit et al., 2020). Other hallmarks of *H. pylori* infection involve the expression of two heat-hock proteins (Hsps), GroES-like HspA (Hsp10) and GroEL-like HspB (Hsp60). The Hsps help to protect against the harsh gastric milieu (Roncarati and Scarlato, 2018; Sit et al., 2020). After adhering to the gastric mucosa, *H. pylori* begins to release additional virulence factors, which can damage host cells either directly or by inducing inflammatory responses (Couturier, 2013). The neutrophil activating protein (NAP) in *H. pylori* can attract neutrophils, monocytes and dendritic cells into the infected area (Couturier, 2013; Peng et al., 2018). Afterwards, the cytokine IL-10 secreted from dendritic cells can induce T_reg_ cells, which can promote persistent colonization by suppressing inflammatory responses induced by IL-17 (Kabir, 2011; Narayana et al., 2015). Also, the secretion of IL-8 can be triggered by the high level secretion of IL-17, and IL-8 recruits neutrophils into infected regions, reinforcing the stimulation of inflammation induced by *H. pylori* (Kabir, 2011; Narayana et al., 2015). During inflammation, pro-inflammatory cytokines such as IL-1β, TNF-α, IL-8, IL-6 and IL-17 are upregulated (Kabir, 2011). Interestingly, *H. pylori* demonstrates resistance against attack by these immune cells even if it is recognized by them (Zhao et al., 2014). Finally, several protein/toxin co-factors such as CagA and vacuolating cytotoxin A (VacA) can act as hijackers by disturbing vital cell processes (cell cycle progression, cell junction integrity, cell polarity, cytoskeletal structure, and mitogenic gene regulation) (Lima et al., 2011; Couturier, 2013). In addition, other virulence factors such as duodenal ulcer promoting A (DupA) and outer inflammatory protein A (OipA) are involved in pathogenesis, as inducers of inflammatory response during the colonization process (Baker, 2020; El-Sayed et al., 2020). Moreover, it is possible that the morphology of the bacterium can affect its motility, which is known to be an important factor in the virulence of *H. pylori* (Ansari and Yamaoka, 2019). It appears that helical-shaped *H. pylori* bacteria have faster motility than those with precoccoidal or coccoidal morphology, therefore, bacteria with a helical shape can better prepare themselves to colonize the gastric mucosa, and those bacteria with precoccoidal or coccoidal structures are more easily cleared from the gastric mucosa (Worku et al., 2015; Salama, 2020).

## Antibiotic-Based Treatment of *H. pylori* Infection

### Current Clinical Treatment Based on Polypharmacy

The Scottish-born scientist, Sir Alexander Fleming is credited for the discovery of “penicillin” as the first antibiotic in 1923, and along with Howard Florey has been called the “father of the antibiotic era” (Gaynes, 2017; Liu et al., 2019). Nowadays, antibiotics are extensively used by clinicians throughout the world to treat bacterial infections (Xiong et al., 2014). Currently, the conventional antibiotics generally utilized in the treatment of *H. pylori* infection include, clarithromycin, metronidazole, amoxicillin, levofloxacin, tetracycline and rifampicin (Arora et al., 2012; Katzung, 2012; Xiong et al., 2014; Talebi Bezmin Abadi, 2017). A simple glance at the current treatment recommendations shows that mono-antibiotic therapy to fight *H. pylori* infection is not recommended by gastroenterologists, due to instability in the gastric environment, low bioavailability, and the short half-life of these drugs (Talebi Bezmin Abadi, 2014; Xiong et al., 2014; Talebi Bezmin Abadi, 2017). To improve therapeutic efficacy, antibiotics can be combined with proton pump inhibitors (PPIs) to reduce acid secretion during first-line triple and second-line quadruple antibiotic regimens (Talebi Bezmin Abadi, 2014; Fallone et al., 2019). Some well-known PPIs are, omeprazole, rabeprazole and lansoprazole (Arora et al., 2012). The beneficial effects of these drugs is ascribed to increasing the pH of the stomach to protect the antibiotic structure within the acidic environment, which leads to increased stability, better tissue penetration, and absorption of the antibiotics (Arora et al., 2012). Although many investigations have tended to use conventional PPIs for *H. pylori* eradication (Talebi Bezmin Abadi, 2014; Oppong et al., 2015), the unwanted condition of atrophic gastritis can emerge after long-term consumption of PPIs (Hagiwara et al., 2015). In a very recent review study, Talebi Bezmin Abadi and Ierardi evaluated the literature before February 2019 concerning the application of vonoprazan as a novel acid-blocker, which could be a suitable alternative to conventional PPIs (Abadi and Ierardi, 2019). The authors confirmed that vonoprazan could potentiate the onset of action of antibiotics, while showing, safety, tolerability, and effective inhibition of gastric acid secretion without any undesirable side effects. The authors suggested that new standard antibiotic regimens containing vonoprazan could be used for future treatment of *H. pylori* infection.

Conventionally, the term “polypharmacy” refers to concurrent use of five (or more than five) separate drugs in adults, which can often produce unfavorable drug-drug interactions (Frahm et al., 2019; Payne, 2020; Sugioka et al., 2020; Turnbull et al., 2020; Weng et al., 2020). In 2018, Bakaki et al. (2018) suggested that this term could also be defined as the prescription of two (or more than two) drugs to pediatric patients. Over recent decades, data obtained from clinical studies has proved that polypharmacy based on triple combination therapy is a suitable regimen for *H. pylori* eradication. It consists of a PPI (standard dose), clarithromycin (500 mg), and amoxicillin (1 g) over 10–14 days treatment course (Katzung, 2006; Talebi Bezmin Abadi, 2016). Since the first isolation of *H. pylori* from human gastric tissue, the overall cure rate using this regimen has consistently decreased. Therefore, clinicians have used a quadruple therapy regiment for the same 14 days as a treatment course. Quadruple therapy consists of standard dose of PPI twice a day, metronidazole 500 mg three times daily, bismuth subsalicylate 525 mg, and tetracycline 250–500 mg four times daily (Katzung, 2012). Saracino’s group suggested that 10 days of bismuth-based quadruple therapy under the name of “Pylera^®^” which contains in one capsule, 140 mg bismuth subcitrate, 125 mg metronidazole, 125 mg tetracycline, prescribed as three tablets four times a day, along with esomeprazole 20 mg twice day could be useful for eradication of *H. pylori*. In this study, the Pylera^®^ regimen was administered to 153 patients with an overall cure rate of 88.3% (Saracino et al., 2020).

Additionally, it has been proposed that polypharmacy based on a non-bismuth quadruple regimen could be also used when bismuth is not available, which was called concomitant therapy (Gisbert and Calvet, 2011; Talebi Bezmin Abadi, 2014; Wang et al., 2017). This regimen includes PPI (standard dose), amoxicillin (1 g), metronidazole (500 mg), and clarithromycin (500 mg) (Gisbert and Calvet, 2011; Talebi Bezmin Abadi, 2014; Wang et al., 2017). Indeed, triple therapy becomes concomitant therapy when 500 mg of metronidazole or tinidazole is added twice daily to improve therapeutic efficacy (Gisbert and Calvet, 2011; Molina-Infante and Gisbert, 2014; Talebi Bezmin Abadi, 2014; Wang et al., 2017). Recent innovations led to development of polypharmacy based on sequential therapy (as an alternative to triple therapy) which includes PPI (standard dose) and amoxicillin (1 g) for first 5 days of therapy, and then PPI (standard dose), clarithromycin (500 mg) and metronidazole (500 mg) for the remaining 5 days (Gisbert and Calvet, 2011; Talebi Bezmin Abadi, 2014; Roszczenko-Jasińska et al., 2020).

### Stewardship and Routes of Antibiotic Administration

The pharmacy literature has shown that specific routes of drug administration, such as oral, nasal, vaginal, pulmonary, ocular, and buccal have all been proposed for individual applications (Bae and Park, 2020). Also, it is known that oral administration of antibiotics can have more long term problems than other routes of administration, such as parenteral and Intravenous (IV) (Kelly et al., 2020). Despite the problems associated with oral administration of antibiotics, oral antibiotic therapy has been traditionally prescribed by clinicians due to the need for good patient compliance, its cost-effectiveness, non-invasiveness, and ease of use (Ahadian et al., 2020; Laffleur and Keckeis, 2020). Moreover, the intraluminal pathway has been used to decrease the duration of treatment as well as boosting the antibiotic activity. Liou et al. (2019) used three types of antibiotics, including a single-dose regimen of amoxicillin (3 g), metronidazole (2 g) and crushed enteric-coated clarithromycin (1 g) which were mixed with a sucralfate suspension for intraluminal administration. They reported that the eradication rate of *H. pylori* with standard triple therapy plus intraluminal administration of antibiotics was 87%, higher than that for 1-week standard triple therapy which was 81.2%.

## Biopharmaceutical Principles of Antibacterial Agents

### Pharmacokinetics and Pharmacodynamics (PK/PD)

Although considerable efforts have been made to discover or design novel candidate antibiotics, most of them are never approved due to poor biopharmaceutical performance. A review by Bae and Park (2020) discussed the biopharmaceutical aspects of biomaterials. They critically asked an important question: “*If a capable scientist makes a new polymer that can treat cancer in humans, isn*’*t it required for the scientist to know the basic pharmacokinetics or how drugs work in humans?*” The answer is clearly “yes, it is urgently required for both conventional drugs and new biomaterials”. In 2019, the global antibiotic research and development partnership (GARD) held a workshop to evaluate the role of PK and PD in determining the required dosage regimens to prevent the antibiotic resistance crisis of pathogens (Theuretzbacher et al., 2020). Thus, it is of the utmost importance to understand the general concepts and biopharmaceutical principles of antibacterial agents (Bergman et al., 2007). The term “PK” is defined as the time course of drug movement within the body (Neely and Reed, 2018; Khoruts et al., 2021). The main factors in PK methodology are known as ADME (absorption, distribution, metabolism, and excretion) (Bergman et al., 2007; Asín-Prieto et al., 2015; Yılmaz and Özcengiz, 2017; Neely and Reed, 2018; Udy et al., 2018). The term “PD” is defined as the pharmacological properties of a drug within the body (Bergman et al., 2007; Khoruts et al., 2021). In systemic absorption, in order to reach the bloodstream, the drug must remain intact when it passes through the stomach, resist metabolism, and avoid liver excretion (Bergman et al., 2007; Neely and Reed, 2018). The term “bioavailability” is defined as the amount of drug absorbed into the bloodstream. It is calculated as:

$$F=\frac{AUC_{Oral}}{AUC_{IV}}$$ (1)

The parameters F , $AUC_{Oral}$ and $AUC_{IV}$ ( AUC :Area under curve) represent drug bioavailability, dose-corrected area under curve for oral and IV routes, respectively (Neely and Reed, 2018).

The term AUC refers to the amount of exposure to a drug and also its elimination from the body. This value can be calculated from a plot of plasma concentration of a drug versus time (Scheff et al., 2011). Moreover, the term “bacterial bioavailability” is defined as the amount of substance per colony forming unit (CFU) (Ropponen et al., 2021). The major PK concepts regarding bacterial bioavailability include uptake, distribution, metabolism, and efflux (Ropponen et al., 2021). In an excellent review by Ropponen et al. (2021), the authors discussed assays to predict antibiotic accumulation in different compartments of bacteria. For an in-depth understanding of these assays, interested readers are referred to the above-mentioned work.

IV drug administration does not require drug absorption from the gastrointestinal tract (GIT) to show therapeutic efficacy (Neely and Reed, 2018). Drugs with lower molecular weight have better absorption than drugs with higher molecular weight (Bergman et al., 2007). Also, drugs with higher solubility and permeability will show better systematic absorption (Neely and Reed, 2018). After absorption of the drug in the body, it will distribute to various tissues (Bergman et al., 2007; Neely and Reed, 2018). Biodistribution refers to the drug spreading through the blood circulation into all the accessible anatomic compartments of the body. The best biodistribution occurs for small, non-protein-bound, non-ionized, lipid-soluble drugs (Bergman et al., 2007; Neely and Reed, 2018). Moreover, factors including age, diet, lifestyle, and diseases can influence the biodistribution (Neely and Reed, 2018). The key parameter in biodistribution is the volume of distribution ( $V_{d}$ ) which is calculated by using the following formulas:

$$V_{d}=\frac{Dose}{C_{P}}$$ (2)

**FIGURE 5:**
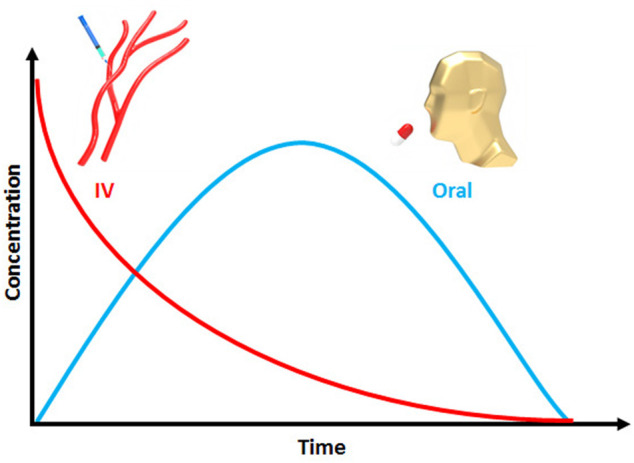
Plasma drug concentration against time for oral and IV administrations.

Where, $C_{p}$ is the peak plasma drug concentration (Neely and Reed, 2018). The most common processes in drug metabolism are oxidation, hydrolysis and reduction. These processes are catalyzed by the activity of cytochrome P-450 enzymes which are present in the liver and kidneys (Bergman et al., 2007; Khoruts et al., 2021). The drug clearance evaluates drug removal from the body per unit time, or in volume per time (Neely and Reed, 2018). It can occur by renal or non-renal pathways (Bergman et al., 2007; Neely and Reed, 2018). The clearance ( Cl ) is calculated as follows:

$$Cl=\frac{Dose\times F}{AUC}\;\mathrm{or}\;Cl=\frac{0.693\times V_{d}}{T_{1/2}}$$ (3)

where $T_{1/2}$ is the drug elimination half-life (Neely and Reed, 2018). Figure 5 schematically shows PK profiles of IV and oral routes of antibiotic administration. The PD concept considers interactions between drugs and biological factors, including enzymes or proteins, to determine drug efficacy and toxicity (Neely and Reed, 2018; Khoruts et al., 2021). These interactions can be agonism or antagonism to various receptors (Neely and Reed, 2018; Khoruts et al., 2021). In receptor agonism, the important point is the activation of receptors via inherent pharmacological effects of a drug. Compared with receptor agonism, the opposite is receptor antagonism, where the drug inhibits the receptor activity (Lista and Sirimaturos, 2021).

### Pharmaceutical Microbiology Concepts

**FIGURE 6:**
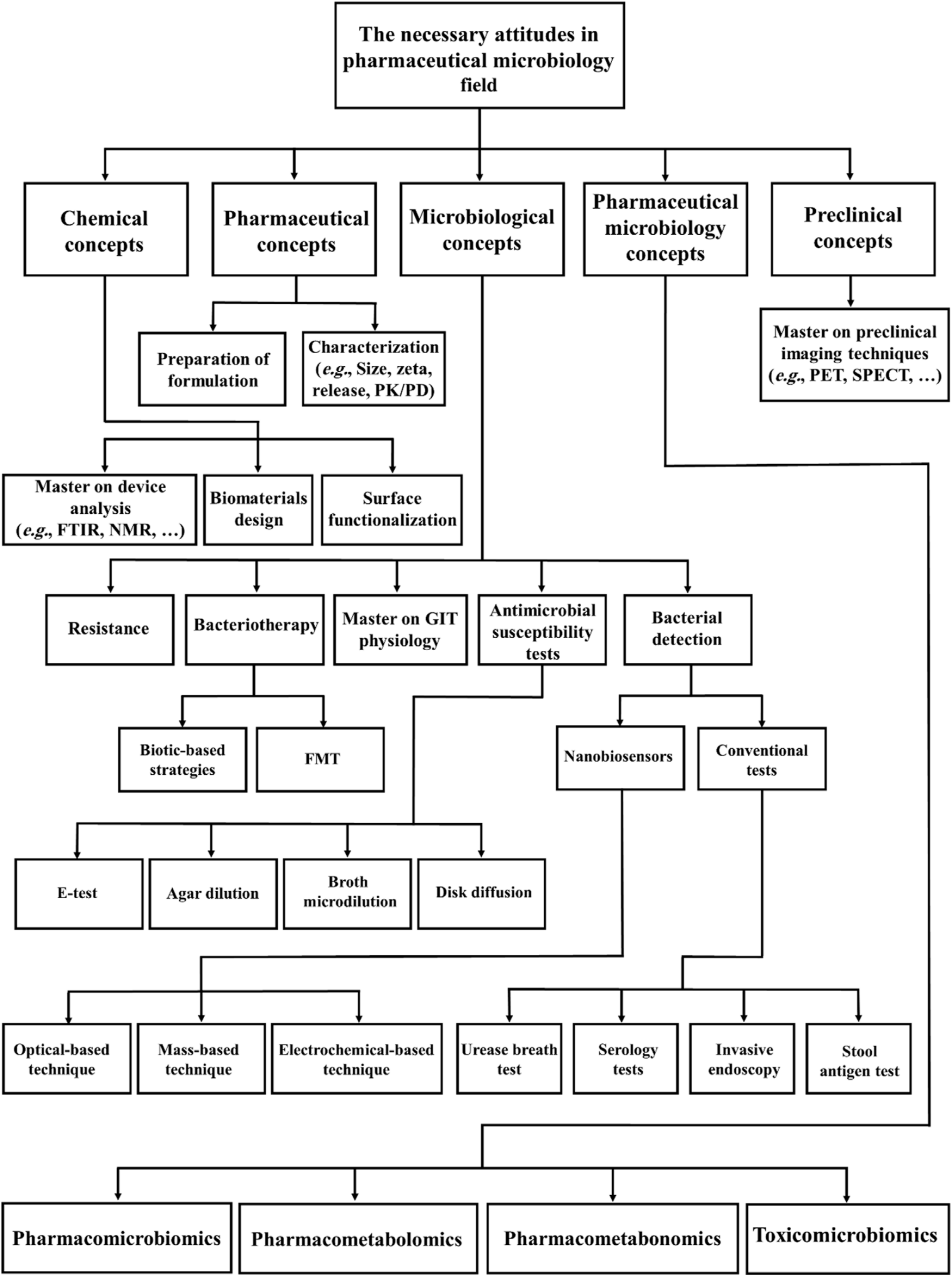
Schematic representation of concepts in pharmaceutical microbiology relevant to the treatment of bacterial infections (FTIR: Fourier Transform Infrared Spectroscopy, NMR: Nuclear Magnetic Resonance, PET: Positron emission tomography, SPECT: Single photon emission computed tomography, PK/PD: Pharmacokinetics/Pharmacodynamics, GIT: Gastrointestinal tract and FMT: Fecal microbiota transplantation).

In modern microbiological research, the recent development of pharmaceutical products based on microorganisms represents an emerging branch of pharmacy and microbiology namely “pharmaceutical microbiology” (Sandle, 2016; Li et al., 2020). In 2016, Sandle described the potential of field to produce new pharmaceuticals based on microbiological concepts (Sandle, 2016). According to Magdy (2014) the main role of a microbiologist in pharmaceutical companies is to assure the safety and sterility of raw biomaterials before manufacturing. However other major concepts in pharmaceutical microbiology can be exploited not only for treatment of *H. pylori* infection, but also for other recalcitrant bacterial infections (Figure 6).

Before discussing in detail the major concepts of pharmaceutical microbiology, it is necessary to review information on the microbiota. The term “microbiota” is defined as a collection of microbial communities which live on or in a particular biological system (Abdelsalam et al., 2020). The term was preceded by the use of “flora” or “microflora” (Abdelsalam et al., 2020). The gut microbiota is also considered as a virtual organ in modern biological science (Carlucci et al., 2016). The normal human gut microbiota is composed of harmless microorganisms ranging from Gram-positive *Firmicutes* to several Gram-negative *Bacteroidetes* such as *Bacteroides*, *Prevotella*, *Parabacteroides*, and *Alistipes* (Roy and Trinchieri, 2017; Toor et al., 2019). Their normal functions include the control of host metabolism, nutrition, homeostasis, inflammation, and immunity. An appropriate balance between these microbes is pivotal to keep the human body in a healthy condition (Dzutsev et al., 2015; Roy and Trinchieri, 2017; Visconti et al., 2019). Importantly, the composition of host gut microbiota can be affected by metabolic, inflammatory bowel disease, physiological, and immune-related diseases (Roy and Trinchieri, 2017; Chung et al., 2018). In order to choose a good animal model for microbiota research, a review by Zheng et al. (2018) suggested that honey bees could be appropriate because of similarities between honey bee gut microbiota and human gut microbiota. Such research can provide an understanding of microbe-microbe and microbe-host interactions, which take place in the human body.

Moreover, a great deal of attention has recently been paid to pharmacogenomics, and this has also led to a surge of interest in emerging pharmacomicrobiomics (Weersma et al., 2020). This term was first introduced by Rizkallah et al. (2010). While pharmacogenomics concentrates on interactions between drugs and the human genome, pharmacomicrobiomics focuses on interactions between drugs and the host gut microbiota, which can influence the PK/PD of pharmaceuticals (Aziz et al., 2018; Doestzada et al., 2018; Sharma et al., 2019; Scher et al., 2020). Given the lack of specific pharmacogenomics and pharmacomicrobiomics testing for many drugs, Aziz et al. (2020) asked the question: *“Has not the time come for routine microbiome testing and establishing pharmacomicrobiomic guidelines, at least for some drugs, in 2020?”*. Therefore there is a strong need to determine clinical guidelines, at least for those drugs which have well-identified pharmacomicrobiomic interactions (Aziz et al., 2020). Equally important is the fact that the association between microbial metabolites and drug responses should be further investigated (Sharma et al., 2019). This is applicable in two related fields, pharmacometabolomics and pharmacometabonomics (Sharma et al., 2019). The major point in pharmacometabolomics is the metabotypes resulting from environmental, chemical, genetic, and physiological factors, while pharmacometabonomics involves the measurement of metabolic profiles in bio-fluids (Everett, 2015; Balasopoulou et al., 2016). For the detection of such metabolites, mass spectrometry and NMR spectroscopy are mainly used for analyzing metabolic data (Everett, 2015; Van Der Hooft et al., 2020). Another term used in pharmaceutical microbiology is toxicomicrobiomics. This involves the interaction between host gut microbiota and xenobiotics (foods, drugs, and environmental chemicals) in terms of toxicology (Aziz, 2018; Abdelsalam et al., 2020). Some of these interactions may increase or decrease the toxicity of antibiotics. Thus, these interactions should be comprehensively explored in pharmaceutical microbiology. To date, the use of pharmaceutical microbiology concepts is still lacking in many studies. If mechanistic data in this field can be more widely appreciated, many of the challenges associated with antibiotic treatment could be addressed.

## Relationship Between Biopharmaceutical Principles of Antibiotics and *H. pylori* Eradication

The antibacterial properties of a drug are strongly dependent on the affinity of the pharmaceutical agent to the targeted microbes, and provision of a sufficient drug dose into the infected area (Neely and Reed, 2018). According to microbiological *in vitro* tests, the minimum inhibitory concentration (MIC) and minimum bactericidal concentration (MBC) values, are the gold standards for determining the biocidal activity of pharmaceuticals (Bergman et al., 2007; Feng et al., 2019). As its name suggests, MIC is the lowest concentration of an antibiotic to inhibit bacterial growth, whereas MBC is lowest concentration of an antibiotic to kill a certain number of logs of a particular bacterium (Andrews, 2001). Many efforts have been made to overcome the limitations of bacterial eradication, by an accurate selection of the PK/PD properties of pharmaceuticals. Antibiotics are generally divided into two categories, time-dependent antibiotics (beta-lactams, tetracyclines, clindamycin and macrolides, except for azalides such as azithromycin) and concentration-dependent antibiotics (such as vancomycin, aminoglycosides, azithromycin, ketolides, and quinolones) (Bergman et al., 2007; Pelgrift and Friedman, 2013; Neely and Reed, 2018). The PK/PD ratio is the major factor used to evaluate the efficacy of antibiotics: T_max_:MIC, C_max_:MIC, and AUC :MIC, where T_max_ is the required time to reach maximum drug concentration and C_max_ is the maximum drug concentration in plasma (Bergman et al., 2007; Pelgrift and Friedman, 2013; Neely and Reed, 2018; Rao and Landersdorfer, 2021). For time-dependent antibiotics, when T_max_ > MIC, the bacteria will be largely killed, and antibiotic resistance cannot easily develop. For concentration-dependent antibiotics, when C_max_:MIC and AUC :MIC are above than a threshold value, bacterial eradication can occur (Bergman et al., 2007; Pelgrift and Friedman, 2013; Neely and Reed, 2018). A meta-analysis study by Chen et al. (2017) showed that the AUC :MIC ratio for amoxicillin in the PPI/clarithromycin/amoxicillin regimen was equal to 5.66. This value led to eradication of *H. pylori*. In another study, Feng et al. (2019) combined the parameters including mutant prevention concentration (MPC) and mutant selection window (MSW) with the PK/PD ratio. MPC is the lowest concentration of an antibiotic that prevents a mutant resistant subpopulation from emerging in a large bacterial population, usually more than 10^10^ CFU/ml bacteria. MSW is an antibiotic concentration above the MIC and below the MPC. The MPC parameter can explain why the rate of clarithromycin resistance is increased. MPC was combined with PK/PD values for low dose (200 mg bid) and high dose (500 mg bid) of clarithromycin to explain variations in MIC values. The authors used T_max_:MIC, C_max_:MIC, AUC :MIC, T_max_:MPC, C_max_:MPC and AUC :MPC ratios to understand the efficacy of clarithromycin for *H. pylori* eradication. They concluded that a low dosage of clarithromycin might lead to either clarithromycin sensitivity or to clarithromycin resistance.

## Newly Proposed Candidate Drugs and Regimens for Treatment of *H. pylori*

There is an urgent need to discover new alternative antibiotics for rescuing clinical failures. A study by Andrei et al. (2019) pointed out that the number of FDA-approved antibacterial drugs (FDA: United.States. Food and Drug Administration) was strikingly lower than drugs for other indications, over more than a quarter-century. In one study, Jin et al. (2018) suggested that standard triple therapy containing ilaprazole could be used for the future treatment of *H. pylori*. They showed that there was no significant change in PK drug interactions. Although they reported the PK parameters of ilaprazole, including AUC and T_1/2_ were slightly higher, while the value of C_max_ was slightly lower when ilaprazole was administered with clarithromycin and amoxicillin, the difference was not statistically significant. On the other hand, Cao et al. (2012) concluded that there was a significant decrease in the values of C_max_ and AUC of ilaprazole and ilaprazole sulfone in the triple combination, while there was no clinically significant PK drug interaction between drugs when ilaprazole thiol ether was included in the regimen. Moreover, it is important to consider whether sex or ethnicity can have any effects on PK drug interactions (Jin et al., 2018). Undoubtedly, this subject necessitates further clinical studies. Recently, a variety of novel antimicrobial drugs and regimens have been proposed by researchers. Some of these anti-*H. pylori* biomaterials are shown in Table 1. A brief look at the current literature shows that biopharmaceutical principles (PK/PD indices and pharmaceutical microbiology concepts) of the many already known anti-*H. pylori* agents and regimens have been neglected so far. Also, in order to simplify and make general conclusions toward improved management of *H. pylori* infection, it is clear that we still have to much learn about the biopharmaceutical implications which are still in their infancy. With these points in mind, we propose that these novel insights could be utilized to improve antibacterial efficiency of novel *H. pylori* inhibitors.

**TABLE 1:** Newly proposed antimicrobial agents and regimens to eradicate *H. pylori* infection.

Newly proposed candidate drugs and regimens	Type of study	Anti-*H. pylori* efficacy	References
Armeniaspirol A	*In vitro*	MIC = 8 μg/ml	Jia et al. (2022)
Armeniaspirol A and omeprazole	*In vivo*	99.56% inhibition of bacterial load.	Jia et al. (2022)
Y4K15 actinomycetes	*In vitro*	The zone of inhibition was 14.9 ± 0.7 mm.	Risandiansyah and Yahya (2022)
Fucoidan	*In vitro/in vivo*	• Inhibition of bacterial colonization to 40% at 2000 μg/ml of fucoidan.	Chen et al. (2022)
		• Reduction of *in vivo* bacterial colonization and pro-inflammatory cytokines (*e.g*, TNF-α and IL-6)	
Amino alcohol xanthone derivatives	*In vitro*	The lowest MIC value was 20 mg/ml against clarithromycin and metronidazole resistant strains.	Klesiewicz et al. (2016)
5-aminoisobenzofuran-1(3H)-one derivatives	*In vitro*	≥70% inhibition of Inosine 5′-monophosphate dehydrogenase (a prokaryotic enzyme) at concentration of 10 μM.	Shah et al. (2019)
^a^cis-[Ru(NO_2_) (bpy)_2_(5NIM)]PF_6_	*In vitro*	• MIC = 512 μg/ml	Sasahara et al. (2020)
		• MBC = 1,024 μg/ml	
Chrysin plus clarithromycin	*In vitro*	Significant reduction of the MIC value of clarithromycin (reported as up to 8 times).	González et al. (2019)
Hesperetin plus metronidazole	*In vitro*	Significant reduction of the MIC value of metronidazole (reported as up to 16 times).	González et al. (2019)
^b^Furazolidone in combination with traditional therapy	*In vivo*	• The intention-to-treat eradication rates were 78.5%, 81.1% and 82% for OAB-M-F, OAC-P and OAB-C-F groups, respectively.	Riahizadeh et al. (2010)
		• The Per- protocol eradication rates were 91.3%, 90.4% and 88.7% for OAB-M-F, OAC-P and OAB-C-F groups, respectively.	
Resveratrol (RSV) and its phenol derivatives (RSV1 to RSV5)	*In vitro/in vivo*	Appearance of higher antimicrobial activity for RSV3 (MIC = 6.25–200 μg/ml) and RSV4 (MIC = 3.12–200 μg/ml).	Di Fermo et al. (2020)
Rabeprazole (20 mg) plus amoxicillin (1 g)	*In vivo*	Emergence of similar pharmacological activities for both suggested high dose dual therapy (eradication rate: 89.6%) and bismuth-based quadruple therapy (eradication rate: 91.2%)	Shao et al. (2022)
Polysorbate 80 (350 mg) in combination with clarithromycin (500 mg), metronidazole (500 mg) and omeprazole (20 mg)	*In vivo*	Enhancement of permeability of antibiotics into the *H. pylori* membrane by polysorbate 80.	Di Stefano et al. (2019)
^c^ *Saccharomyces boulardii* or *Lactobacillus reuteri* plus 14 days traditional quadruple therapy	*In vivo*	The eradication rates were reported as 94.2% and 92.3%, respectively.	Naghibzadeh et al. (2022)
^d^ *Lactobacillus reuteri* plus 10 days sequential therapy or 7 days standard triple therapy	*In vivo*	The eradication rates were reported as 88% and 63%, respectively.	Efrati et al. (2012)

a bpy, 2,2′-bipyridine; 5-NIM, 5-nitroimidazole.

b OAB-M-F; metronidazole (M) (500 mg bid) for the first 5 days, followed by furazolidone (F) (200 mg bid) for the second 5 days, OAC-P; clarithromycin (C) (500 mg bid) for 10 days; and OAB-C-F; clarithromycin (500 mg bid) for the first 5 days and furazolidone (200 mg bid) for the second 5 days.

c *Saccharomyces boulardii* and *Lactobacillus reuteri* are beneficial microbes (so-called as probiotics) used against *H. pylori*.

d These anti-*H. pylori* agents could be co-administered with traditional antibiotic therapy to increase the eradication rate.

## Future Perspectives and Conclusion

Over the years, *H. pylori* has been discovered to be as major causative agent of a variety of gastric diseases such as peptic ulcer, gastric ulcer and gastric cancer. As stated earlier, triple and quadruple antibiotic therapy regimens are by far the preferred option for clinicians. Statistical analysis from recent microbiological studies has confirmed that the antibiotic resistance crisis is becoming critical for healthcare, and this also applies to *H. pylori*. Due to the sky-rocketing development of antibiotic resistance, there is an ever-increasing need to improve current clinical approaches. If nothing changes, we will probably encounter a new pandemic of extremely resistant bacteria in the near future. To overcome the challenges associated with antibiotic therapy, it is necessary to consider biopharmaceutical principles of antibiotics such as PK/PD studies and concepts of pharmaceutical microbiology. Despite undeniable challenges, there is still a hope for improving current antibiotic therapy in the near future.
